# Effects of Different Environmental Factors on the Growth and Bioactive Substance Accumulation of *Porphyridium purpureum*

**DOI:** 10.3390/ijerph17072221

**Published:** 2020-03-26

**Authors:** Xudan Lu, Fangru Nan, Jia Feng, Junping Lv, Qi Liu, Xudong Liu, Shulian Xie

**Affiliations:** School of Life Science, Shanxi University, Taiyuan 030006, China; luxd9999@163.com (X.L.); nanfr@sxu.edu.cn (F.N.); fengj@sxu.edu.cn (J.F.); lvjunping024@sxu.edu.cn (J.L.); liuqi@sxu.edu.cn (Q.L.); liuxudong@sxu.edu.cn (X.L.)

**Keywords:** *Porphyridium purpureum*, environmental factors, growth, bioactive substance accumulation

## Abstract

Genus *Porphyridium* is a primitive single-celled red algae widely distributed in seawater, freshwater, and moist soil. It can synthesize bioactive substances such as phycoerythrin, extracellular polysaccharides and polyunsaturated fatty acids during the growth process. In this paper, the culture and bioactive substance yield of *Porphyridium purpureum* were studied by setting salinity, nitrogen-to-phosphorus ratio, and pH at different gradient levels. The results showed that the optimal conditions for the growth of *P. purpureum* were salinity 34 ppt, nitrogen-to-phosphorus ratio 169:1, and pH 8; the optimal conditions for obtaining the polysaccharides were salinity 17 ppt, nitrogen-to-phosphorus ratio 14:1, and pH 8; the optimal conditions for obtaining phycoerythrin were salinity 17 ppt, nitrogen-to-phosphorus ratio 68:1, and pH 8; the optimal conditions for obtaining the lipids were salinity 34 ppt, nitrogen-to-phosphorus ratio 1:1, and pH 8. In actual production applications, culture conditions should be set according to different product accumulation purposes in order to achieve the optimal production efficiency.

## 1. Introduction

As the world’s population and energy demand increase, there is a key issue in finding new renewable energy sources to produce energy. As the most promising source of biomass in biofuel production, microalgae has the most outstanding advantages of high growth rate and high photosynthetic rate in specific environments [1], which is 10 times more capable of fixing CO_2_ than terrestrial plants, and 30–100 times more efficient energically per hectare than crops [2]. In addition, as a biofuel, microalgae also has the advantages of not occupying cultivated land, not affected by the season, high yield, and no lignin in the cell wall, which is conducive to the production of bioethanol [3]. Microalgae are autotrophic organisms that use light energy and inorganic nutrients (carbon dioxide, nitrogen, phosphorus, etc.) to synthesize valuable biomass compounds such as lipids, proteins, carbohydrates, pigments, etc. They not only provide good raw materials for the production of bio-ethanol and bio-petroleum, but also can be widely used in food, pharmaceutical, and cosmetic industries [4,5].

*Porphyridium purpureum (*Rhodophyta, Porphyridiophyceae, Porphyridiales, Porphyridiaceae, *Porphyridium*), which is a relatively primitive single-celled red algae with high salt tolerance, can synthesize valuable bioactive substances such as phycoerythrin, extracellular polysaccharides, and polyunsaturated fatty acids during the growth process. Therefore, it is getting more and more attention [6]. Most of the proteins in the *Porphyridium* are phycobiliproteins, including phycoerythrin, phycocyanin, and allophycocyanin, of which phycoerythrin is the main phycobiliprotein and also the reason why the cells are fuchsia. The phycobiliprotein can be used as a natural pigment in food, cosmetics, dyes, and other industries, and can also be made into fluorescent reagents for clinical medical diagnosis, immunochemistry, bioengineering research, food, and medicine for medical healthcare [7]. In addition, *Porphyridium* polysaccharides are another high-value compound, wrapped on the cell wall surface in the form of sulfated polysaccharidess. The external part of the polysaccharides is dissolved from the cell surface into the medium during the liquid culture, increasing the viscoelasticity of the medium [8], and the unique rheological property allows them to be used as lubricants and thickeners, etc. [9]. Research by Dvir et al. has shown that *Porphyridium* polysaccharides have nutritional, medicinal, and cosmetic values [10]. Specially, the polysaccharide extracted from *Porphyridium* sp. showed antiviral activities and was more effective in inhibiting retrovirus replication and cell transformation by MuSV (murine sarcoma virus) [11,12,13]. The lipids in *Porphyridium* are stored mainly in the form of triacylglycerol (TAG), which is transesterified to produce biodiesel and to provide energy for metabolic processes [14]. And some long-chain fatty acids (LC-PUFAs) have high medical value, such as eicosapentaenoic acid (EPA, C20: 5) that reduces the risk of diabetes, brain disease, inflammation, arteriosclerosis, heart disease, and several cancers, and arachidonic acid (ARA, C20: 4), which also has basic functions in the treatment of cardiovascular disease, rheumatoid arthritis, and cancer [15].

Since the 1960s, scholars have researched and made good progress in taxonomy, ecology, nutrition, cultivation, and application of *Porphyridium* [16]. According to existing studies, microalgae can respond to changing environmental conditions by regulating their metabolites [14], so that changes in environmental conditions can be utilized to achieve a large accumulation of required bioactive substances. The main environmental factors affecting the growth of microalgae are nitrogen and phosphorus concentration, salinity, temperature, light, and pH. Among them, nitrogen and phosphorus are essential nutrients for algae growth, used to synthesize chlorophyll and other photosynthetic pigments, amino acids, nucleic acids, coenzymes, and phospholipids [16], and salinity, as an important factor affecting the growth of microalgae, will also affect the accumulation of microalgae fatty acids and carbohydrates [17]. Therefore, the use of different environmental factors to achieve the large-scale accumulation of required bioactive substances has become the key to *Porphyridium* research. In recent years, research on the culture conditions of *Porphyridium* has been relatively rare, and previous literatures only focused on the effects of a certain culture condition on one bioactive product [18,19,20]. However, the responses to different environmental factors of the most valuable bioactive substances in *Porphyridium,* including phycoerythrin, polysaccharides and fatty acids, have not been systematically studied.

The purpose of this study was to systematically study the growth and metabolism of *P. purpureum* at different salinities, nitrogen-to-phosphorus ratios, and pH. The effects of different salinity, nitrogen-to-phosphorus ratio, and pH on the algal growth characteristics and the unit accumulation of polysaccharides, phycoerythrin, and total lipids were obtained. The novelty of this study is evidenced by clarifying the optimal culture conditions (salinity, nitrogen-to-phosphorus ratio and pH) for growth and bioactive substance yields (polysaccharides, phycoerythrin, and total lipids) of *P. purpureum*. It provides basis for large-scale cultivation, production, and application of *P. purpureum* in future research. 

## 2. Materials and Methods 

### 2.1. Strains and Culture

The original species of *Porphyridium purpureum* was purchased from FACHB (Freshwater Algae Culture Collection at the Institute of Hydrobiology, Wuhan, China), and the algae species number was FACHB-806. Meanwhile, the Kock medium was obtained from the website of FACHB [21]. The main components and volume of the medium are shown in Table 1, and artificial seawater components are shown in Table 2. In addition, the soil extract was configured as follows: 200 g garden soil without fertilizer and 1000 mL dH_2_O were put in a triangle bottle with a breathable stopper, the mixture was heated in water bath at 100 °C for 3 h and then precipitated for 24 h, and this process was performed three times in a row. Then, the mixture was filtered and the supernatant was sterilized in an autoclave and stored in a 4 °C refrigerator for later use.

### 2.2. Different Growth Condition Treatments

Based on the principle of controlling a single variable, we set the salinity, nitrogen-to-phosphorus ratio, and pH at different gradient levels by adjusting the amount of related components in the KOCK medium. The salinity of natural seawater is about 34 ppt. We set four salinity gradients of 0 ppt, 17 ppt, 34 ppt, and 68 ppt by adjusting the amount of seawater components in the medium. With KOCK medium with KNO_3_ as the nitrogen source, the content was 0.05, 0.25, 1.0, and 1.25 g/L. With KH_2_PO_4_ as the phosphorus source, the content was 0.07, 0.025, 0.02, and 0.01 g/L in order; the four nitrogen-to-phosphorus ratio gradients were set to 1:1, 14:1, 68:1, and 169:1. We set three gradients of pH 6, pH 7, and pH 8 by adjusting the initial pH of the medium.

*P. purpureum* grown to logarithmic phase was inoculated into a 250 mL erlenmeyer flask containing 200 mL KOCK medium at an initial concentration of about 2 x 10^4^ cells/ml. The mixture was then cultured in a light−dark period of 12 h/12 h, the illumination was 60 μmol/m^2^/sec, and the culture temperature was (25 ± 2) °C. The algal solution was shaken about three times a day.

### 2.3. Determination of Algae Growth

Photomicrographs of *P. purpureum* cells under different growth conditions were taken using an optical microscope, and the effects of different environmental factors on the morphology and growth of *P. purpureum* cells were obtained.

Micro count of *P. purpureum* cells was conducted by using a cytometry plate every three days, and then the growth curves of *P. purpureum* under different conditions were drawn.

### 2.4. Determination of Chlorophyll Fluorescence

After storing 3 mL algal suspension in the dark for 30 min, two PSII (photosystem II) indexes of chlorophyll fluorescence were determined according to [22] by the portable PAM fluorometer (AquaPen-C AC100, Prague, Czech). The two indexes included the maximum quantum yield of photosystem II photochemistry (Fv/Fm) and the potential activity of photosystem II (Fv/F0).

### 2.5. Determination of Polysaccharides Content

First, 20 mL of algal suspension was taken and centrifuged at 6000 rpm for 10 minutes, the supernatant was discarded, the algae was freeze-dried for 24 hto obtain the algal powder, and the mass of algal powder was recorded as m_0_. Then, 30 mL dH_2_O was added into the 100 mL beaker with the algal powder, the mixture was concentrated in a water bath at 80 °C to about one-third of the original volume, and the final volume was recorded as V. Next, the mixture was centrifuged at 6000 rpm for 5 min, and the supernatant was taken as the crude polysaccharides solution. Finally, polysaccharides concentration was determined according to the phenol-sulfuric acid method [23], that was, 5 mL concentrated sulfuric acid and 1 mL 6% phenol were added to a test tube with 1 mL polysaccharides solution, the mixture was heated in a water bath at 100 °C for 15 min, and the absorbance value was measured at 490 nm after cooling. The polysaccharides concentration, C_0,_ was calculated based on a standard curve, which drawn based on a 40 μg/ml glucose standard solution [24]. The polysaccharides content (PC) was calculated according to the following formula:

PC(%) = C_0_V/m_0_ × 100% (1)

### 2.6. Determination of Phycoerythrin Content

First, 20 mL of algal suspension was lyophilized into algal powder after a series of treatments, and the mass of the algal powder was recorded as m_0_. Subsequently, the algal powder was suspended with 5 mL phosphate buffer solution (10 mmol/L, pH 7.0). Next, the suspension was frozen in −80 °C refrigerator for 1 hour and thawed in water bath at 37 °C, this process was repeated three times, and then the algal cell was broken by an ultrasonic crusher (SCIENTZ-IID, Scientz, Ningbo, China) at 20% (at the rated power) for 5 min. Then, the suspension was centrifuged at 6000 rpm, 4 °C for 5 min, and the supernatant was saved. Finally, the absorbance value was measured at 595 nm according to the Bradford method [25], and then the phycoerythrin concentration, C_0,_ was calculated according to the standard curve, which was drawn based on a 100 μg/ml BSA (bovine serum albumin) standard solution [26]. The phycoerythrin content (RC) was calculated according to the following formula:

RC(%)=5 C_0_/ m_0_×100% (2)

### 2.7. Determination of Total Lipids Content

Total lipids content was determined gravimetrically according to the modified Bligh and Dyer methods [27]. First, the algal cultivation broth in the stationary phase was lyophilized into algae powder after a series of treatments, and the mass of algae powder was recorded as m_0_. Subsequently, 2 mL chloroform and 4 mL methanol were added into the glass bottle with the algal powder, and the mixture was placed in an ultrasonic crusher to break up cells. Next, the mixture was centrifuged at 5000 rpm for 5 minutes, the supernatant was collected in a new glass bottle, the mass of the empty glass bottle was recorded as m_1_. The above steps were repeated at least three times. Then, 1% NaCl solution was added into the collected supernatant until the layer was separated, and the lower layer was saved. Finally, the lipids solution in the glass bottle was dried under nitrogen flow, and the mass of the glass bottle with the lipids was recorded as m_2_ [28]. The total lipids content (LC) was calculated according to the following formula:

LC(%) = (m_2_ − m_1_)/ m_0_ × 100% (3)

### 2.8. Statistical Analysis

The experiment was replicated three times. All data were expressed as mean ± standard error. One-way ANOVA was used to test the significance of different environmental factors on the growth and organic matter accumulation of *P. purpureum* (*p* ≤ 0.05). All statistical analyses were carried out using the SPSS19.0 statistical software (IBM Inc. Chicago, IL, USA).

## 3. Results

### 3.1. Algal Growth

#### 3.1.1. Microscopic Observation

**Figure 1:**
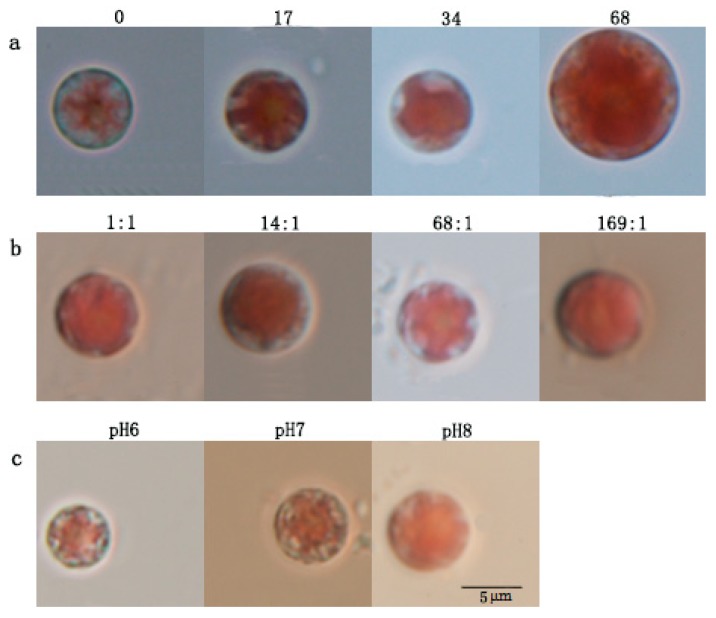
Cell morphology of *P. purpureum* under different conditions; the photos were taken under a 100× optical microscope. (**a**). Cell morphology of *P. purpureum* at different salinities. (**b**). Cell morphology of *P. purpureum* at different nitrogen-to-phosphorus ratios. (**c**). Cell morphology of *P. purpureum* at different pH conditions.

As shown in Figure 1a, when the salinity of medium was 0 ppt (fresh water conditions), the growth status of *P. purpureum* was the worst, the cell vitality was the weakest, and the pigment bodies were star-shaped, but the color was lighter. The shape of pigment bodies became irregular as salinity was raised. The diameter of *P. purpureum* cells was the largest at salinity 68 ppt. As shown in Figure 1b, the size of a single *P. purpureum* cell increased as the nitrogen-to-phosphorus ratio was raised. The pigment bodies showed a star shape at nitrogen-to-phosphorus ratio 68: 1 but showed an irregular shape at other nitrogen-to-phosphorus ratios. As shown in Figure 1c, the cell vitality was relatively weak and the pigment bodies shrank at pH 6 and pH 7, but *P. purpureum* cells were more vigorous at pH 8. The cell size showed an increasing trend with the increase of pH.

#### 3.1.2. Growth Curve

**Figure 2:**
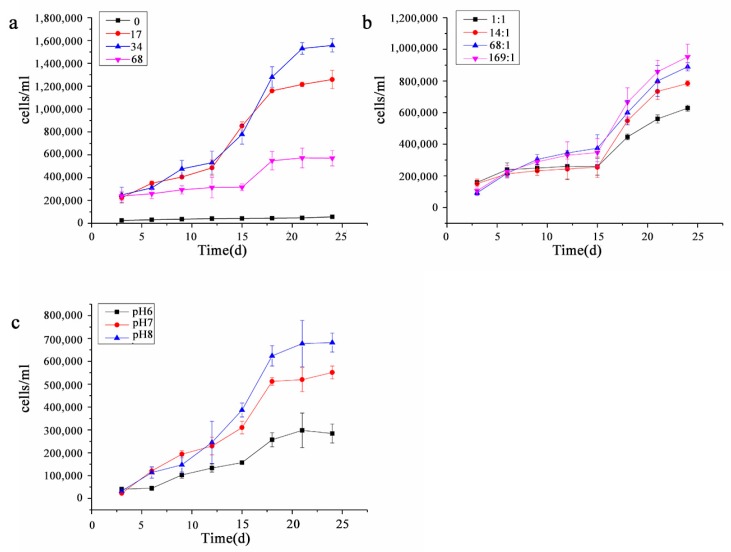
Growth curves of *P. purpureum* under different environmental factors. Data were expressed as mean ± standard error. (**a**). The growth curves of *P. purpureum* at different salinities. (**b**). The growth curves of *P. purpureum* at different nitrogen to phosphorus ratios. (**c**). The growth curves of *P. purpureum* at different pH conditions.

As shown in Figure 2, *P. purpureum* entered the stationary phase on the 17th day under different salinities and pH conditions and on the 20th day under different nitrogen-to-phosphorus ratios, but the effects on the growth rate and cell density of *P. purpureum* were different under different environmental factors. As shown in Figure 2a, the cell density in the stationary phase at different salinities was 34 ppt > 17 ppt > 68 ppt > 0 ppt in order. *P. purpureum* showed the same growth trend and similar cell density in the logarithmic growth phase at salinity 17 ppt and 34 ppt. The growth was milder and the growth rate was lower at salinity 0 ppt than in the other salinity experiment group, and *P. purpureum* could still grow normally and reach a high cell density of 1,571,500 cells/ml at salinity 68 ppt (high-salinity conditions). As shown in Figure 2b, the cell density in the stationary phase at different nitrogen-to-phosphorus ratios was 169:1 > 68:1 > 14:1 > 1:1 in order, and it showed an increasing trend with the increase of the nitrogen-to-phosphorus ratio. As shown in Figure 2c, the cell density in the stationary phase at different pH was pH 8 > pH 7 > pH 6 in order. *P. purpureum* grew faster in the prologarithmic phase at pH 7, but grew faster and started to exhibit the highest cell density in the postlogarithmic phase at pH 8.

### 3.2. Analysis of Chlorophyll Fluorescence

**Figure 3:**
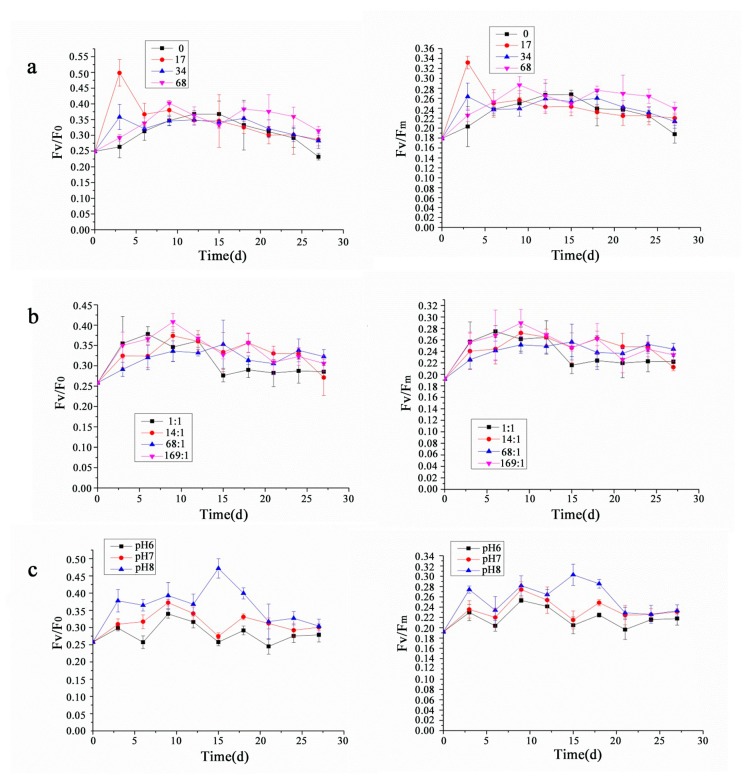
The chlorophyll fluorescence of *P. purpureum* under different environmental factors. Data were expressed as mean ± standard error. (**a**). The chlorophyll fluorescence of *P. purpureum* at different salinities. (**b**). The chlorophyll fluorescence of *P. purpureum* at different nitrogen-to-phosphorus ratios. (**c**). The chlorophyll fluorescence of *P. purpureum* at different pH conditions.

As shown in Figure 3, the chlorophyll fluorescence parameters, Fv/ Fo and Fv/ Fm, showed variable trends under different conditions. As shown in Figure 3a, the chlorophyll fluorescence parameters were the largest at salinity 17 ppt from 0 to 6 days, followed by that at salinity 34 ppt and 68 ppt, with the smallest at salinity 0 ppt (fresh water conditions). The chlorophyll fluorescence parameters showed a steady trend from 7 to 18 days. However, they showed a slow downward trend after 19 days, the chlorophyll fluorescence parameters were the largest at salinity 68 ppt, followed by those at salinity 34 ppt and 17 ppt, with the smallest at salinity 0 ppt, and there was no significant difference in chlorophyll fluorescence parameters under different salinities. As shown in Figure 3b, the chlorophyll fluorescence parameters at different nitrogen-to-phosphorus ratios showed an upward trend from 0 to 10 days. However, they began to show a slow downward trend after 11 days, and they were always the smallest at the nitrogen-to-phosphorus ratio 1:1. As shown in Figure 3c, the chlorophyll fluorescence parameters gradually stabilized after 21 days, and they were always the highest at pH 8, followed by those at pH 7, and the smallest at pH 6.

### 3.3. Contents of Polysaccharides

**Figure 4:**
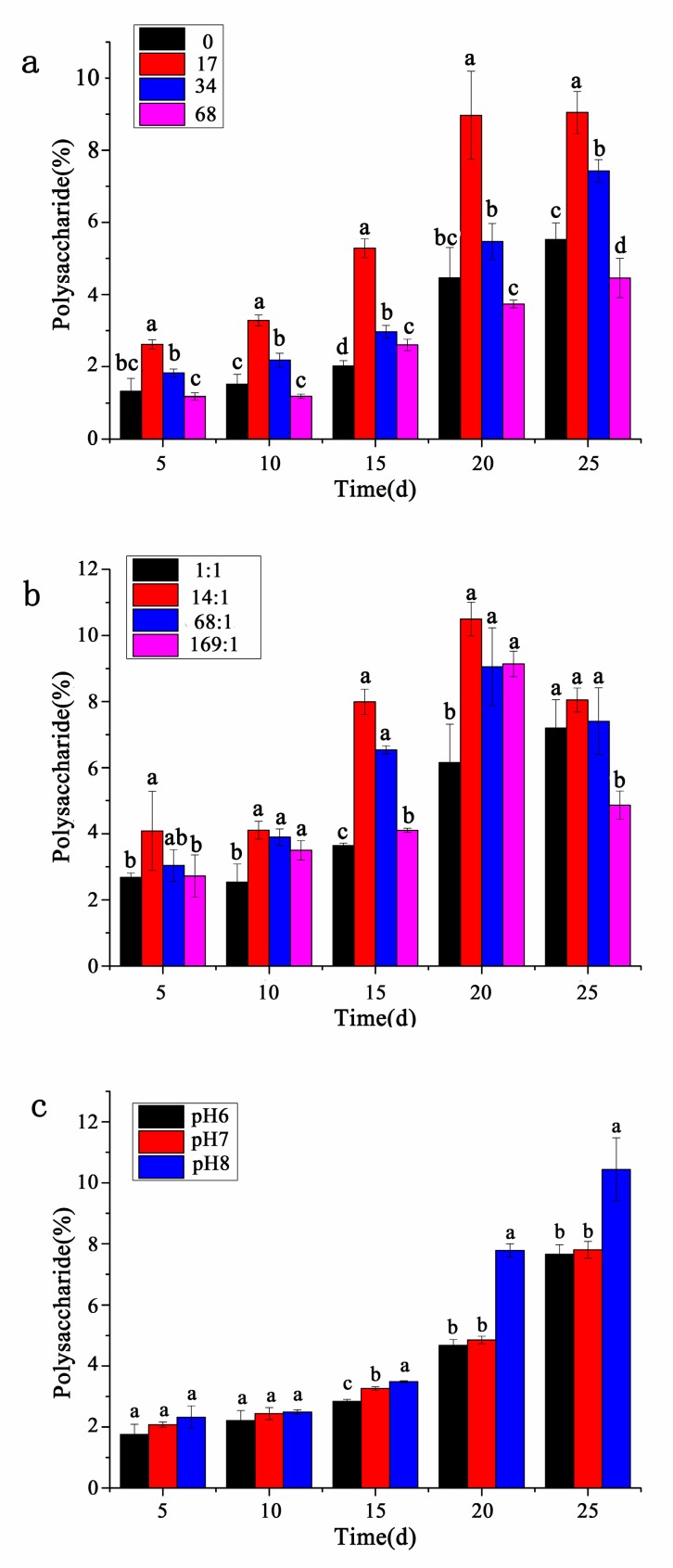
The content of polysaccharides of *P. purpureum* under different environmental factors. Data were expressed as mean ± standard error. (**a**). *P. purpureum* polysaccharides content at different salinity. (**b**). *P. purpureum* polysaccharides content at different nitrogen-to-phosphorus ratios. (**c**). *P. purpureum* polysaccharides content at different pH conditions.

As shown in Figure 4, the content of polysaccharides per unit algal powder under different conditions gradually increased with time, and it began to accumulate in large quantities especially in the postlogarithmic phase. Therefore, the total amount of polysaccharides had a significant positive correlation with the cell growth. 

As shown in Figure 4a, the accumulation of polysaccharides at different salinities reached the maximum on the 25th day, and polysaccharides contents at salinity 0 and 68 were relatively lower, respectively 5.52% and 4.46%, 7.42% at salinity 34 ppt, the highest at salinity 17 ppt, which was to 9.05%, and there were significant differences between polysaccharides contents under different salinities. As shown in Figure 4b, the polysaccharides content at different nitrogen-to-phosphorus ratios (except 1:1) showed a slight downward trend on the 25th day. The polysaccharides content was the largest at nitrogen-to-phosphorus ratio 14:1 compared with other groups from the day of inoculation to the stationary phase, especially on the 20th day, which was 10.50%, and there was significant difference in polysaccharides content between nitrogen-to-phosphorus ratio 1:1 and other groups. As shown in Figure 4c, from the day of inoculation to the stationary growth phase, the polysaccharides content was always the highest at pH 8, followed by that at pH 7, with the lowest at pH 6, and the maximum accumulation at different pH was reached on the 25th day, 10.44%, 7.81%, and 7.66%, respectively, and there was a significant difference between the polysaccharides content at pH 8 and other groups.

### 3.4. Contents of Phycoerythrin

**Figure 5:**
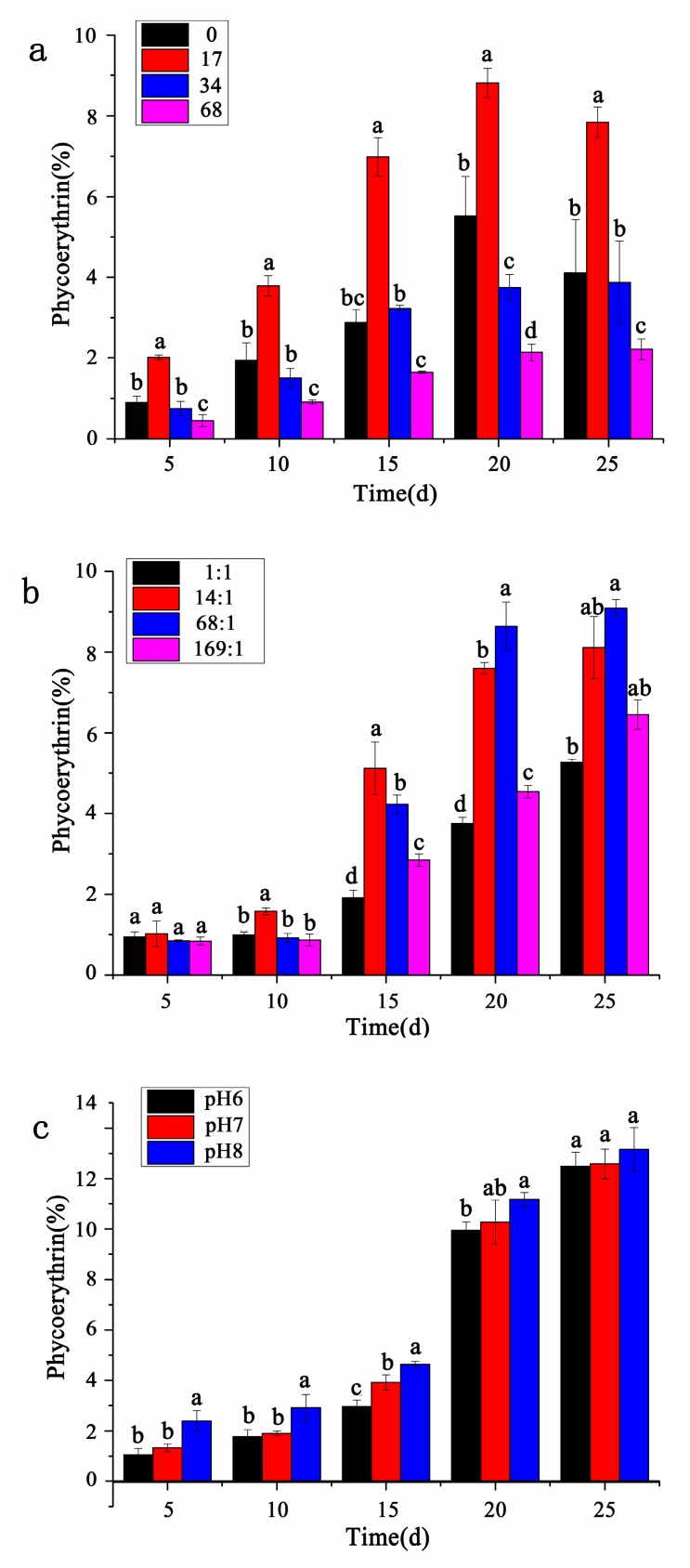
The content of phycoerythrin of *P. purpureum* under different environmental factors. Data were expressed as mean ± standard error. (**a**). The content of phycoerythrin of *P. purpureum* at different salinities. (**b**). The content of phycoerythrin of *P. purpureum* at different nitrogen-to-phosphorus ratios. (**c**). The content of phycoerythrin of *P. purpureum* at different pH conditions.

As shown in Figure 5, the content of phycoerythrin per unit algal powder under different conditions gradually increased with time, and it began to accumulate in large quantities especially in the postlogarithmic phase. Therefore, the total amount of phycoerythrin had a significant positive correlation with the cell growth. However, there was a difference that the phycoerythrin content at different salinities showed a slight downward trend on the 25th day (Figure 5a). In addition, the accumulation of phycoerythrin at different salinities reached the maximum on the 20th day, and the phycoerythrin contents at salinity 0 ppt, 34 ppt, and 68 ppt were relatively low, respectively 5.52%, 3.75%, and 2.14%, highest at salinity 17 ppt, with a content of 8.81%, and there were significant differences between the phycoerythrin contents at different salinities.

As shown in Figure 5b, the phycoerythrin content was always highest at nitrogen-to-phosphorus ratio 14:1 from 0 to 15 days (before reaching the stationary phase), but was the highest at nitrogen-to-phosphorus ratio 68:1 after entering the stationary growth phase. On the 25th day, the content at nitrogen-to-phosphorus ratio 68:1 was 9.09%, there was only a significant difference between the phycoerythrin content at nitrogen-to-phosphorus ratio 68:1 and nitrogen-to-phosphorus ratio 1:1. On the 20th day, the content at nitrogen-to-phosphorus ratio 68:1 was 8.64%, however, there were significant differences between the phycoerythrin content at different nitrogen-to-phosphorus ratios. As shown in Figure 5c, from 0 to 25 days, the phycoerythrin content was always the highest at pH 8, followed by that at pH 7, with the lowest at pH 6, and the accumulation of phycoerythrin at different pH reached the maximum on the 25th day, 13.16%, 12.58%, and 12.50%, respectively, and there was no significant difference between the phycoerythrin content at different pH.

### 3.5. Contents of Total Lipids

**Figure 6:**
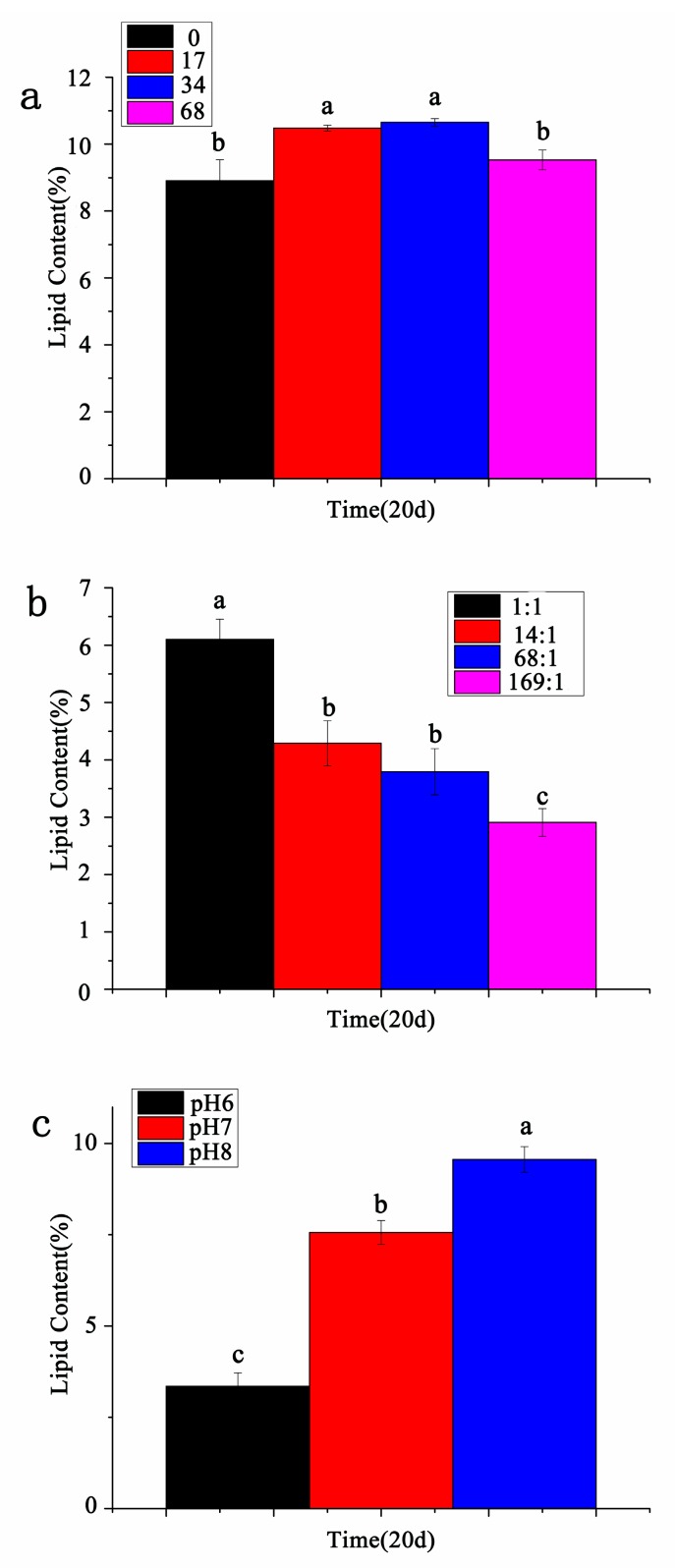
The content of total lipids of *P. purpureum* under different environmental factors. Data were expressed as mean ± standard error. (**a**). *P. purpureum* total lipid content under different salinity; (**b**). *P. purpureum* total lipid content under different N:P ratios; (**c**). *P. purpureum* total lipid content under different pH conditions.

As shown in Figure 6a, on the 20th day, the total lipid content was highest at salinity 34 ppt, with a content of 10.65%, the contents at 17 ppt, 68 ppt, and 0 ppt were relatively low, respectively 10.48%, 9.53%, and 8.91%. There was no significant difference in the total lipid content between salinity 0 ppt and 68 ppt, and there was no significant difference in the total lipid content between salinity 17 ppt and 34 ppt.

As shown in Figure 6b, on the 20th day, the total lipid content of *P. purpureum* at different nitrogen-to-phosphorus ratios was 1:1 > 14:1 > 68:1 > 169:1 in order, which were 6.10%, 4.29%, 3.79%, and 2.91%, respectively. There was only no significant difference in total lipid content between nitrogen-to-phosphorus ratio 14:1 and 68:1, but there were significant differences among the other nitrogen and phosphorus ratio experimental groups. As shown in Figure 6c, on the 20th day, the total lipid content of *P. purpureum* at different pH was pH 8 > pH 7 > pH 6 in order, which were 9.56%, 7.56%, and 3.35%, respectively, and there were significant differences in the total lipid content at different pH.

## 4. Discussion

The growth and metabolism of *P. purpureum* under different environmental factors were studied in this paper. The experimental results showed that *P. purpureum* grew slowly under conditions of excessively high or low salinity, especially in fresh water, where the cell viability was weak and the growth rate was low. Previous studies had shown that the competitive strength of *Porphyridium* was relatively weak when the salt concentration was less than 3.5%, and there was no inhibitory effect on its growth with the salt concentration up to 4.6% [29]. In addition, the growth of *Porphyridium* in seawater was relatively faster compared with freshwater in the early culture period, which was similar to our results [30]. It was reported that *Porphyridium* in seawater began to decline and *Porphyridium* in freshwater began to grow in the later period, and the logarithmic phase and survival time of *Porphyridium* in freshwater were prolonged [30]. Therefore, we can achieve the production purpose of obtaining a high concentration of algae through phased cultivation. Seawater culture can be used to rapidly grow and accumulate *P. purpureum* cells in initial cultivation, and freshwater culture can be used to prolong the survival time of the cells and make biomass continue to increase and accumulate in the later cultivation.

The content of polysaccharides and phycoerythrin of *P. purpureum* cells was highest at salinity 17 ppt and it showed a downward trend as the salinity was raised, which indicated that the low-salt environment was beneficial to the accumulation of soluble polysaccharides and phycoerythrin. It is possible that Na^+^ and Mg^2+^ compete for the ribosome in a high-salt environment to inhibit protein synthesis [31]. In addition, the content of total lipid of *P. purpureum* was the highest at salinity 34 ppt, an indication that salt stress was beneficial to the accumulation of lipid to a certain extent, which was consistent with the report of *Porphyridium* [32]. According to the report, salt stress could enhance the accumulation of lipid in various microalgae, which may be due to the response of microalgae to salt stress through the accumulation of compatible solutes [33]. Therefore, low-salt environment can be exploited to accumulate polysaccharides, and high-salt stress can be used to achieve large-scale synthesis of lipid, which provides good raw materials and methods for the mass production of bioethanol and biodiesel.

The Redfeld ratio (C:N:P = 106:16:1 atomic ratio) [34] has long been used as the optimal nitrogen-to-phosphorus ratio for the growth of phytoplankton, but different phytoplankton have different needs for nutrients in their growth process. In this paper, the biomass accumulation of *P. purpureum* in the stationary phase showed an upward trend with the increase of the nitrogen-to-phosphorus ratio. The cell concentration in the stationary growth phase was highest at nitrogen-to-phosphorus ratio 169:1, which was much higher than the optimal nitrogen-to-phosphorus ratio 16:1 for algae, but lowest at nitrogen-to-phosphorus ratio 1:1, indicating that the growth rate and cell concentration of *P. purpureum* increased when the nitrogen source was excessive and decreased when the nitrogen source was limited, which was consistent with previous research [35]. Studies have shown that the microalgae tended to decrease the rate of growth and photosynthesis, and also tended to degrade nitrogen-containing macromolecules such as proteins to store a large amount of carbon and energy-rich compounds in order to resist the adverse environment, so there was a large accumulation of carbohydrates and lipids under nitrogen starvation [3,36]. In this paper, the content of total lipid of *P. purpureum* was highest at nitrogen-to-phosphorus ratio 1:1, the content of polysaccharides was highest at the nitrogen-to-phosphorus ratio 14:1, and the content of phycoerythrin was highest at nitrogen-to-phosphorus ratio 68:1 (high-nitrogen environment). The low-nitrogen environment was not conducive to protein accumulation but was conducive to the accumulation of polysaccharides and lipid, which was consistent with existing reports. The content of lipid and carbohydrate is significantly increased in various microalgae species under nitrogen starvation, eventually the biofuel production potential of microalgae is improved, but nutritional starvation resulted in reduced biomass and reduced biofuel production. To overcome this problem, researchers have proposed a two-phase culture strategy in which microalgae first grow under sufficient nutrition to produce sufficient biomass, and then grow under nitrogen limitation to produce abundant carbohydrate and lipid [37].

The effect of pH on the growth of *Porphyridium* was mainly manifested in changing the permeability of the cell membrane and the activity of intracellular enzymes, eventually the entry and exit of nutrients and cell metabolism were affected [16]. Previous studies have shown that *Porphyridium* can grow normally at pH 5.0~8.3 [38], and its optimum pH growth range was pH 7.5~8.0 [16], which was similar to our result that the optimal condition for *P. purpureum* growth was at pH 8.0. And the result that the content of phycoerythrin was highest at pH 8 was consistent with our study, but the result that pH 5 was the optimal environment for obtaining extracellular polysaccharides content [39,40] was not consistent with our result. We analyze the possible reasons for this. Our experiments were conducted in lab using the species *P. porphyridium*, whereas the results of optimal pH 5 in previous studies were obtained based on outdoor mass cultures or using a different species *P. cruentum* [39,40]. The optimal pH for producing extracellular polysaccharides was related with the experimental environments and algal species. The total lipids content was the lowest at pH 6. The possible reason is that the acidic environment inhibits related enzyme activities during the synthesis of long-chain fatty acids, such as dehydrogenase, oxygenase, and chain elongase, thereby blocking the conversion to long-chain fatty acids [41] and ultimately leading to a relatively low total lipid content.

Comprehensively speaking, culture condition is a very important factor in the cultivation of *P. purpureum* [30]. Therefore, it is of great significance to monitor the effect of different environmental factors (salinity, nitrogen-to-phosphorus ratio, and pH) on the growth and accumulation of bioactive substances of *P. purpureum*. First of all, the cultivation cell number and chlorophyll fluorescence under different conditions were monitored during cultivation, thus the optimal growth conditions and the algal species harvest time can be obtained, which provides data support for the cultivation optimization of *Porphyridium* in actual production. At the same time, the application value of bioactive substances synthesized by *Porphyridium* during the growth process in medicine, food, or other industries makes their maximum amount production a key. Therefore, the optimal combination of conditions for producing bioactive substance were obtained by monitoring their contents under different conditions during cultivation, which provides theoretical support for improving and optimizing the production of bioactive substances, and also provides good materials for industrial production.

## 5. Conclusions

The effects of salinity, nitrogen-to-phosphorus ratio, and pH on the algal growth, polysaccharides, phycoerythrin, and total lipid content of *P. purpureum* were studied. The results showed that the optimal conditions for the growth of *P. purpureum* were salinity 34 ppt, nitrogen-to-phosphorus ratio 169:1, and pH 8. The optimal conditions for obtaining polysaccharides of *P. purpureum* were salinity 17 ppt, nitrogen-to-phosphorus ratio 14:1, and pH 8. The optimal conditions for obtaining phycoerythrin were salinity 17 ppt, nitrogen-to-phosphorus ratio 68:1, and pH 8. The optimal conditions for obtaining lipid were salinity 34 ppt, nitrogen-to-phosphorus ratio 1:1, and pH 8. In addition, the low-salt and low-nitrogen environment were conducive to the accumulation of carbon-rich compounds in the cell, such as polysaccharides and triacylglycerol. Therefore, it is possible to achieve the purpose of accumulating a large amount of bioactive substances by regulating the growth conditions of *P. purpureum*.

## Figures and Tables

**Table 1:** Kock medium.

Components	Volume	Mother Liquor Concentration
KNO_3_	10 mL/L	7.5 g/100ml dH_2_O
KH_2_PO_4_	1 mL/L	2.5 g/100ml dH_2_O
MgSO_4_.7H_2_O	10 mL/L	2.0 g/100ml dH_2_O
Ferric citrate	1 mL/L	0.25 g/100ml dH_2_O
Soil extract	10 mL/L	
dH_2_O	484 mL	
Seawater	484 mL	

**Table 2:** Artificial seawater components (1 L).

Components	Dosage
NaCl	27.0 g
MgSO_4_.7H_2_O	6.6 g
MgCl_2_.6H_2_O	5.6 g
CaCl_2_.2H_2_O	1.5 g
KNO_3_	1.0 g
KH_2_PO_4_	0.07 g
NaHCO_3_	0.04 g
1M Tris-HCl	20 mL
Trace element mother liquor	1 mL
Fe-EDTA (Ethylene Diamine Tetraacetic Acid)	1 mL
